# Geometric Fidelity of Magnetic Resonance Imaging and Computed Tomography-Derived Virtual 3D Models of Porcine Cadaver Mandibles: Conventional Versus Artificial Intelligence-Based Segmentation

**DOI:** 10.3290/j.ohpd.c_2365

**Published:** 2025-11-26

**Authors:** Lucas M. Ritschl, Katharina Pippich, Matthias Herrmann, Herbert Deppe, Anton Sculean, Monika Probst, Florian A. Probst

**Affiliations:** a Lucas M. Ritschl Department of Oral and Maxillofacial Surgery, TUM School of Medicine and Health, Technical University of Munich, Klinikum rechts der Isar, Ismaninger Strasse 22, 81675 Munich, Germany. Study conception and design, analysis and interpretation, main writing and revision of the manuscript.; b Katharina Pippich Department of Oral and Maxillofacial Surgery, TUM School of Medicine and Health, Technical University of Munich, Klinikum rechts der Isar, Ismaninger Strasse 22, 81675 Munich, Germany. Data analysis and interpretation, revision of the manuscript.; c Matthias Herrmann Department of Oral and Maxillofacial Surgery, TUM School of Medicine and Health, Technical University of Munich, Klinikum rechts der Isar, Ismaninger Strasse 22, 81675 Munich, Germany. Data acquisition, analysis and interpretation, revision of the manuscript.; d Herbert Deppe Department of Oral and Maxillofacial Surgery, TUM School of Medicine and Health, Technical University of Munich, Klinikum rechts der Isar, Ismaninger Strasse 22, 81675 Munich, Germany. Study conception and design, revision of the manuscript.; e Anton Sculean Department of Periodontology, School of Dental Medicine, University of Bern, Bern, Switzerland. Study conception and design, revision of the manuscript.; f Monika Probst Department of Diagnostic and Interventional Neuroradiology, TUM School of Medicine and Health, Technical University of Munich, Klinikum rechts der Isar, Ismaninger Strasse 22, 81675 Munich, Germany; MRadenT GmbH, Sendlinger Strasse 37, 80331 Munich, Germany. Study conception and design, revision of the manuscript.; g Florian A. Probst MKG Probst, Sendlinger Strasse 37, 80331 Munich, Germany; Department of Oral and Maxillofacial Surgery and Facial Plastic Surgery, University Hospital, Ludwig Maximilians University, 80337 Munich, Germany. Study conception and design, data acquisition, data analysis and interpretation, and the writing and revision of the manuscript.

**Keywords:** artificial intelligence, computed tomography, computer-assisted surgery, image processing, magnetic resonance imaging, segmentation

## Abstract

**Purpose:**

The workflow for virtual surgical planning (VSP) and the application of CAD/CAM (computer-aided design/computer-aided manufacturing) procedures are mainly based on computed tomography (CT) derived DICOM data sets. Alternatively, this study aims to preclinically illuminate the feasibility of a magnetic resonance imaging (MRI) based workflow and the impact of artificial intelligence (AI) based segmentation on the required fidelity on basic 3D geometry acquisition.

**Materials and Methods:**

Porcine cadaver mandibles were imaged with CT and a T1-weighted MRI sequence. The resulting DICOM data sets were segmented conventionally (Mimics Medical 17.0, Materialize; Belgium) and with AI-based segmentation software (ImFusion Labels and Suite, Version 2.19.2, ImFusion; Germany). The four standard tessellation language (STL) files were superimposed with a corresponding reference model derived from an optic scan (Artec Space Spider, Artec 3D; Luxembourg) and the following parameters were analysed: Hausdorff distance (HD), mean surface distance (MSD), root mean square distance (RMSD), time.

**Results:**

In comparison to the reference model, all four parameters were significantly (P <0.001) better for the CT imaging and the AI-based segmentation. MRI-derived AI-based segmentation reached the fidelity of CT imaging data sets and conventional segmentation (HD, MSD, and RMSD each P >0.05).

**Conclusion:**

The use of AI-based segmentation software proved to be useful and feasible for MRI-derived data sets, and generated the desired 3D geometry more quickly while maintaining the necessary quality. Nevertheless, the results for the CT were still better and remain yet the standard.

Virtual surgical planning (VSP) and the application of CAD/CAM (computer-aided design/computer-aided manufacturing) technology are nowadays the gold standard in cranio-maxillofacial surgery, including reconstructive surgery, traumatology, implantology, and orthognathic surgery. With special regard to reconstructive microsurgery, the application offers for instance shorter surgery and ischaemia times and hospital stays with improved symmetry, aesthetics, bony consolidation rates, and function.^15,31,42,43^ In addition, it was also shown that virtual planning can be reliably translated into the operating room.^28^ But according to the commonly applied digital workflow sequence, the planning is usually based on a digital images and communications in medicine (DICOM) data set of a computed tomography (CT) or cone beam computed tomography (CBCT) scan. As a result, every patient who undergoes CAD/CAM-assisted surgery is currently potentially exposed to additional radiation. However, according to the ALARA principle (as low as reasonably achievable), radiation exposure should be reduced to the lowest possible level in order to keep the risk of radiation-associated consequences as low as possible.

Thus, with regard to facial bony reconstruction, especially of the mandible with alloplastic materials or microvascular bone flaps – using the free fibula, iliac crest, or scapula – radiation exposure is currently inevitable. Despite constant efforts to further reduce the radiation dose,^33,50^ there is a general desire for an alternative solution to obtain a DICOM data set for further processing in the digital workflow – the segmentation and thus the conversion of the DICOM data set into, for example, a standard tessellation language (STL) file. However, the necessary accuracy in the acquisition of the geometry should be ensured within a reasonable time frame. On this topic, it has already been described by others that linear measurements between MRI- and CT-derived 3D surface models correspond well.^6,10,14,19^ Additionally, our research group has recently investigated the possibility of adequately capturing the complex mandibular geometry using a T1-weighted magnetic resonance imaging (MRI) sequence compared to CT or CBCT on the porcine mandible.^24^ The results of this study indicated that the accuracy and reliability of T1-weighted MRI-derived virtual 3D bone surface models were equal to CT and CBCT. But segmentation proved tedious in parts and also took longer than for CT- or CBCT-derived data sets. Consequently, with this follow-up study, we now aimed to investigate the impact of artificial intelligence (AI) based segmentation on accuracy and time required to make the promising use of MRI-based VSP in cranio-maxillofacial surgery more feasible.

## MATERIALS AND METHODS

### Ethical Statement and Animal Welfare

The prospective study was planned and performed according to the STARD guidelines. The Municipal Veterinary Office (city of Munich, Germany) approved the cadaveric study. It was not necessary to obtain approval from the institutional ethics committee of the Technical University of Munich, Klinikum rechts der Isar, because this study was a porcine cadaver study of animals already slaughtered independently of this study.

### Study Design

**Fig 1:**
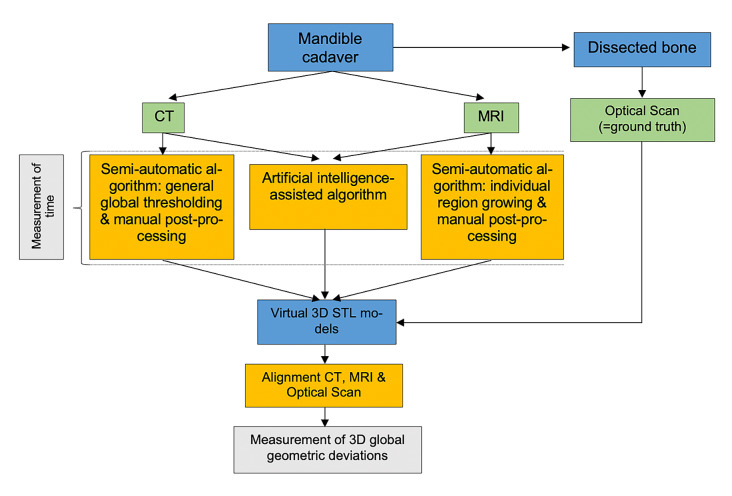
Study design.

This study was planned and performed using the CT- and MRI-derived DICOM data sets, as well as the acquired optical scans of the preliminary study.^24^ But, following segmentation, alignment and analyses were performed completely anew and independently from the preliminary study. The surrounding soft tissue was not removed before imaging (CT or MRI).^24^ One part of the mandibular body (n = 10) served as the region of interest (ROI). The segmentation was done either with a semi-automatic algorithm or an AI-based algorithm derived from CT and MRI images. The segmentation processes were carried out independently by two observers twice (repetition after 6 weeks) for each segmentation algorithm and imaging modality. The resulting mandibular 3D surface geometries were exported as STL files and then compared with corresponding optical scans (= ground truth model) by digitally superimposing them via an alignment procedure (Fig 1).

In order to also examine the time component of the segmentation, the time required for segmentation of the respective lower jaw halves, and to convert the given DICOM data set into an STL file, was recorded.

#### Imaging – computed tomography

The porcine cadaver mandible was aligned with the occlusal plane horizontally to the ground. Then, multi-slice CT images (Philips Ingenuity 128 device, Philips Healthcare; Best, Netherlands) were created with the following parameters: isotropic voxel size 0.67 mm; field of view 20–20 cm; tube voltage 120 kV; tube current-time product 250 mAs.

#### Imaging – magnetic resonance imaging

The porcine cadaver mandibles were placed in a tub that was filled with water. The occlusal plane was aligned approximately horizontally to the floor, and a 16-channel head and neck spine array was placed around the box. The three-dimensional T1-weighted MRI sequence was generated using a 3Tesla MRI system (Elition, Philips Healthcare; Best, Netherlands). This sequence was optimised for bone visualisation and is already in clinical use.^25^ In order to reduce the echo time, partial Fourier imaging in the direction of frequency coding with a factor of 60% was used.^12^ The following parameters were applied to the recordings: Acquisition time 03:08 min; field of view 180 mm; matrix 420 × 419; recorded voxel size 0.6 × 0.6 × 0.6 mm^3^; number of signal averages 1; repetition time 10 ms; echo time 1.55 ms; compressed sensing (CS) + sensitivity encoding (SENSE): yes; reduction factor 2.3.

#### Optical scan of bone surface

After the CT and MRI images were taken, all soft tissue was removed from the porcine cadaver mandibles in order to create an optical three-dimensional scan of the lower jaw (Artec Space Spider, Artec 3D; Luxembourg), which served as a reference (= ground truth model) 47. The cadavers were only processed with conventional dissecting instruments. Neither thermal nor chemical treatment was applied to the bone in order to avoid possible damage to the bone surface and not to falsify the result of the geometric surface comparisons.^11,45^ The manufacturer of the Artec Space Spider scanner specifies a resolution of up to 0.1 mm and a point accuracy of up to 0.05 mm. With the help of the appropriate software (Artec Studio 13, Artec 3D; Luxembourg) and the associated autopilot tool, the data generated in this way were ultimately used to generate three-dimensional scans. In order to use these as a reference and to be able to compare them with the segmentation results, they were exported as an STL file.^24^ The Artec Studio software provides a low maximum error value of 0.5 mm for post-processing scans after fine registration, alignment and global registration.

#### Image processing and segmentation procedures

##### Semi-automatic/conventional algorithm

The conventional semi-automatic algorithm for segmentation was carried out with Mimics (Mimics Medical 17.0, Materialize; Belgium). After opening the Mimics Medical program, the CT- or MRI-derived DICOM data set of the desired porcine cadaver mandible was opened and positioned in the coordinate system. The time measurement was started immediately afterwards.

**Fig 2a and b:**
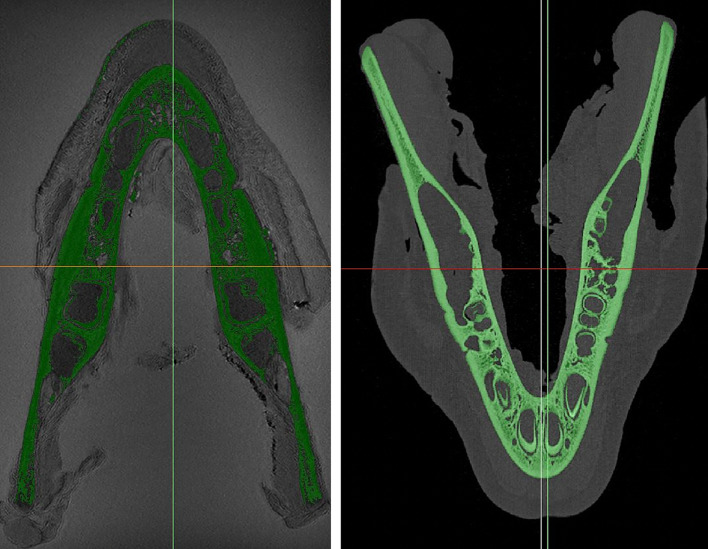
Porcine cadaver mandible after (a) ‘dynamic region growing’ (axial, MRI) and (b) ‘thresholding’ (axial, CT).

The thresholding function was used to segment the CT images (Fig 2), applying the predefined ‘Bone CT’ classification (all voxels with Hounsfield units of 226 and higher). Then the ROI was determined.

The ‘Dynamic Region Growing’ function was used to segment the MRI images. Therefore, all points (seed points) on the jaw were defined, which had assumed values between 0 and 10 GV (grayscale value). The range of ‘Dynamic Region Growing’ was also set between 40 and 50 GV (difference to seed point value) in each case individually. The result of the ‘Dynamic Region Growing’ function is shown in Figure 2.

After thresholding or region growing, manual post-processing of the labels was still necessary for precise segmentation. For this reason, with the help of the ‘Multiple Slice Edit’ tool and the integrated interpolation function, the residual holes and incorrectly marked areas were inspected and repaired manually. Immediately afterwards, the segmented geometry was saved and exported as an STL file, and the time measurement was stopped and noted.

##### Automatic/AI-based algorithm

The AI-based, automatic segmentation was done with the ImFusion Labels and Suite software (Version 2.19.2, ImFusion; Germany). These coherent programs allow the built-in AI to almost completely dispense with any manual post-processing. By appropriately setting a few seed points on individual sectional images of the processed modality, which tell the software where the tissues to be kept apart are located, for example, bone and soft tissue, the automatic algorithm segments the DICOM data across all sectional images and, after a brief check by the observer, converts it into an STL file. The program thus not only provides interpolation between processed sectional images, but also calculates the complete 3D model itself based on the sectional image, without any edge drawing at the tissue boundaries, with only minimal input from the observer.

The following descriptions applied equally to both modalities. After importing and opening the desired DICOM data set, the ImFusion Labels software switched to annotation mode, and time measurement was started. At the beginning of segmentation with this software, no thresholding or region growing was used. The ROI was defined, whereupon the segmentation was started by selecting the ‘Interactive Segmentation’ button.

The settings have now been made on the cursor, which was the main tool for segmentation. First, the size was set to a range between 6 and 20 mm. The second adjustable option was the adaptiveness of the cursor, whereby adaptiveness defined how much the cursor adjusted to the objects. The higher the setting, the more likely it was to protect against drawing beyond the object. Specifically, this meant that while the cursor was being moved over the image, it was shown where the program distinguished the bone from the soft tissue or from the background. However, if the adaptiveness was too high, an overcorrection would result. This overcorrection could largely be avoided with an adaptiveness value between 0.3 and 0.7.

**Fig 3a and b:**
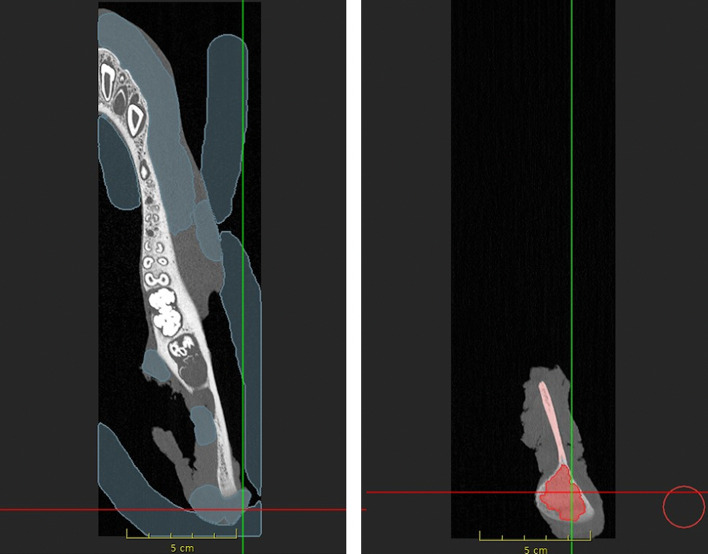
ImFusion Labels in axial layers (a) setting of the background markers and (b) setting of the foreground markers.

In order to begin the desired segmentation, background label seeds were first set (Fig 3a). This was set on a total of five to ten sectional images in the axial section. Then foreground label seeds were placed on the mandibular bone (Fig 3b), also on five to ten layers. Based on these seeds, the software automatically calculated the foreground label completely. Only individual parts of the label had to be corrected by adding additional background and foreground seeds.

After the result was accepted, the program was redirected to ImFusion Suite. After visual inspection of the created 3D geometry, the result was saved and exported as an STL file, and time measurement was stopped and noted.

### Alignment and Cropping

Following, the exported CT- or MRI-derived STL files were aligned and compared to the corresponding optical 3D ground truth model using the software Artec Studio Professional 13 (Artec 3D; Luxembourg). For this purpose, the corresponding STL file was imported and superimposed using the ‘Align’ function. A rigid ICP (iterative closest point) alignment algorithm was employed. The manual rigid alignment procedure was used for this purpose, which only allowed rigid, linear movements and rotations when the objects are superimposed without changes in scale or similar settings 30, based on a three-point alignment. Hence, three corresponding points were selected that were clearly visible and reproducible: the most occlusal tip of the second premolar, the most distal tip of the second molar, and the mental foramen. Based on this manual orientation, the geometries were finally automatically aligned by the software.

To minimise an error in the superimposition measurements, the previously overlaid geometries were ‘cropped’ (trimmed) in Blender (Version 2.82a) to a matching dimension in all three planes. This was to avoid incorrectly high results by measuring incorrect pairs of correlated points with each other.

### Calculation of Geometric Deviations

Based on the alignments, the surface differences between the CT- or MRI-derived STL files and the corresponding optical 3D scans were determined. In order to quantify the differences, we have included key metrics for assessing geometric fidelity, such as the Hausdorff distance (HD),^2,27,49^ mean surface distance (MSD)^30,47^ and root mean square distance (RMSD).^11,29,30,36^ In particular, Hausdorff distance is a well-established method for quantifying the maximum geometric deviation between two-point sets. It considers the greatest deviation within the entire point cloud, rather than just isolated point-pair differences. This provides a more robust and comprehensive analysis of segmentation accuracy, which is clinically more relevant in the setting of CAD/CAM surgical planning. Additionally, pairwise point comparisons offer only fragmented information about the overall geometry of the models.

**Fig 4:**
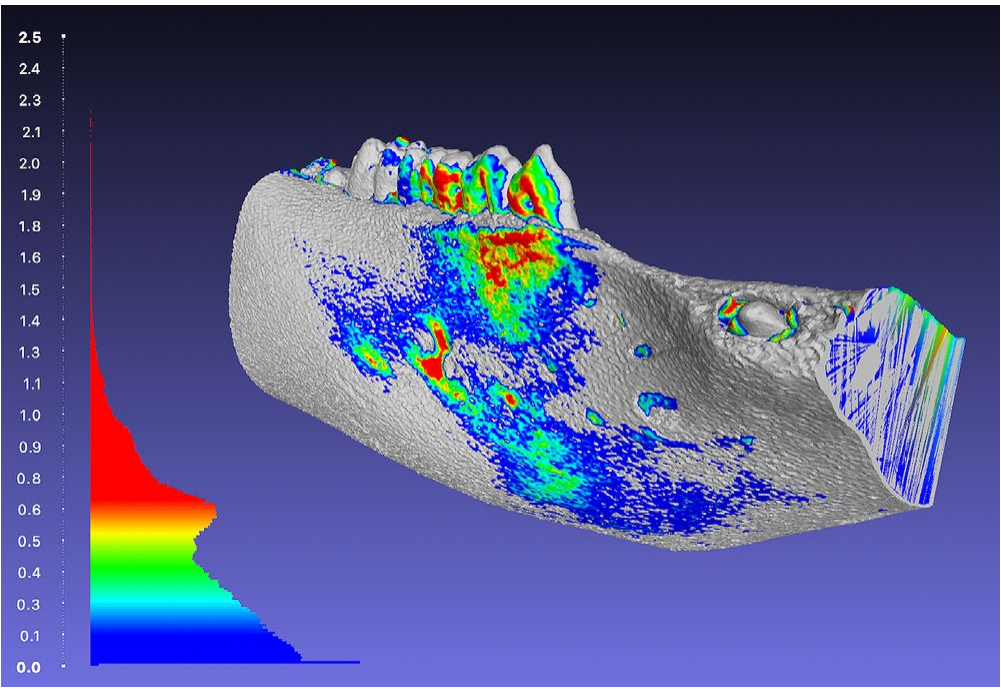
Colour map of the surface deviation measurements [mm] of the segmented porcine cadaver mandibles compared to the optical 3D scan (histogram showing the distributions of the deviations of the corresponding points in mm: red colour indicating higher deviation; blue colour indicating lower deviation; MeshLab (Version 2021.07)).

These measurements were carried out by the open-source software MeshLab (Version 2021.07). In addition, colour maps were used to visualise any geometric deviations (Fig 4).

### Statistics

The intraclass correlation (ICC) coefficient was calculated to determine the intra- and interrater reliability and consistency of measurements performed by two raters applying a two-way mixed model (MH and KP). This was calculated based on the RMSD. As the primary outcome variable, three-dimensional geometric deviations between the given segmentation algorithms or the modalities and the optical 3D scans were set, given as the HD, the MSD, and the RMSD. The time required for segmentation of one half of the jaw based on either algorithm and modality was determined as the secondary outcome variable. Continuous measurements are presented as median (range). In not normally distributed data sets, the Wilcoxon rank sum test was used for dependent samples. All statistical tests were performed on an exploratory two-sided 5% significance level. No adjustments were made for multiple testing. Bland–Altman analyses were performed to additionally assess the agreement of the combinations of the applied segmentation algorithms and modalities with the most common combination, the semi-automatic segmentation of CT data sets. These show their mean differences (MSD values) and upper and lower limits of agreement (mean value of the differences ±1.96 × standard deviation of the differences). Analysis was performed using Excel for Mac software (Microsoft, Redmond, USA) and IBM SPSS 24 for Mac software (IBM, Armonk; New York, USA).

## RESULTS

For the interrater reliability, the ICCs, based on the RMSD, were 0.955 (CT), 0.915 (MRI), 0.933 (semi-automatic), and 0.948 (automatic). Intrarater ICCs were 0.943/0.974 (CT), 0.909/0.928 (MRI), 0.915/0.956 (semi-automatic), and 0.951/0.953 (automatic).

The median geometric deviations of the segmentation results compared to the optical scan were lower for the CT-derived data sets and the automatic segmentation algorithm and are shown in Table 1. These results were highly significant (P < 0.001) for all comparative parameters examined (HD, MSD, and RMSD). A similar result was found for the segmentation time. It was significantly (P <0.001) shorter with CT in comparison to MRI, and with the automatic algorithm in comparison to the semi-automatic algorithm.

**Table 1:** Comparison of modalities and algorithms

	Modality	Algorithm
CT	MRI	Semi-automatic	Automatic
HD [mm]	Count	80	80	80	80
Median	5.282	5.732	5.736	5.153
Minimum	3.117	3.761	3.674	3.117
Maximum	7.308	7.894	7.894	7.304
MSD [mm]	Count	80	80	80	80
Median	0.412	0.466	0.502	0.402
Minimum	0.308	0.365	0.365	0.308
Maximum	0.628	0.752	0.752	0.527
RMSD [mm]	Count	80	80	80	80
Median	0.582	0.679	0.663	0.576
Minimum	0.418	0.515	0.472	0.418
Maximum	0.744	0.851	0.851	0.800
Time [s]	Count	80	80	80	80
Median	848	1504	1321	939
Minimum	505	835	685	505
Maximum	1128	2363	2363	1493


### Combination of Modality and Algorithm

More precisely, the modalities were combined with the segmentation algorithms and compared to standardised procedure: CT imaging with subsequent semi-automatic segmentation.

**Fig 5a to d:**
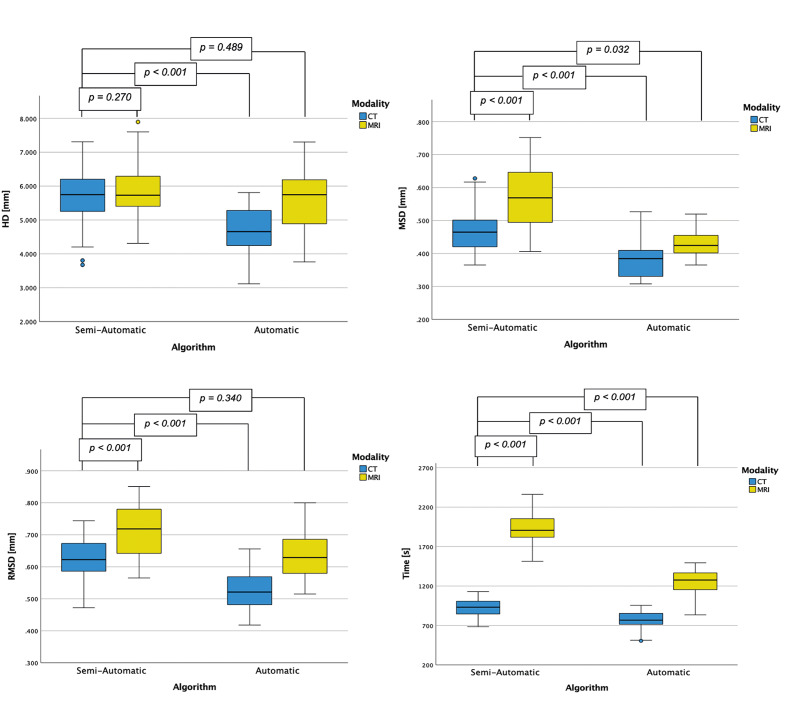
Comparison of different parameters depending on the used modality and the method of segmentation: (a) Hausdorff distances (HD), (b) mean square distances (MSD), (c) root mean square distance (RMSD), and (d) time.

Median geometric deviations of the HD between the segmentation results and the optical scans were 5.746 (3.674– 7.308) mm for the combination of CT/semi-automatic algorithm, 5.732 mm (4.311–7.894) for MRI/semi-automatic algorithm, 4.656 mm (3.117– 5.810) for CT/automatic algorithm, and 5.746 mm (3.761–7.304) for MRI/automatic algorithm. Only the differences between CT/semi-automatic algorithm and CT/automatic algorithm were statistically significant (P <0.001) (Fig 5a).

Median geometric deviations of the MSD between the segmentation results and the optical scans were 0.465 mm (0.365–0.628) for CT/semi-automatic algorithm, 0.569 (0.406–0.752) mm for MRI/semi-automatic algorithm, 0.385 mm (0.308–0.527) for CT/automatic algorithm, and 0.425 mm (0.365–0.520) for MRI/automatic algorithm. Differences between CT/semi-automatic algorithm, MRI/semi-automatic algorithm, and CT/automatic algorithm were all statistically highly significant (P <0.001). Differences between CT/semi-automatic algorithm and MRI/automatic algorithm were also significant (P <0.032) (Fig 5b).

Median geometric deviations with reference to RMSD between the segmentation results and the optical scans were 0.623 mm (0.472–0.744) for CT/semi-automatic algorithm, 0.718 mm (0.565–0.851) for MRI/semi-automatic algorithm, 0.521 mm (0.418–0.656) for CT/automatic algorithm, and 0.629 mm (0.515–0.800) for MRI/automatic algorithm. Differences between CT/semi-automatic algorithm, MRI/semi-automatic algorithm, and CT/automatic algorithm were all statistically highly significant (P <0.001). Differences between CT/semi-automatic algorithm and MRI/automatic algorithm were not significant (P <0.340) (Fig 5c).

Median segmentation times of the examined modalities/algorithms were 932 (685–1128) s for CT/semi-automatic algorithm, 1906 s (1514–2363) for MRI/semi-automatic algorithm, 767 s (505–955) for CT/automatic algorithm, and 1276 s (835–1493) for MRI/automatic algorithm. All time differences based on the combination of the imaging modalities and the segmentation algorithms used, compared with the CT/semi-automatic algorithm, were highly significant (P <0.001) (Fig 5d).

**Fig 6:**
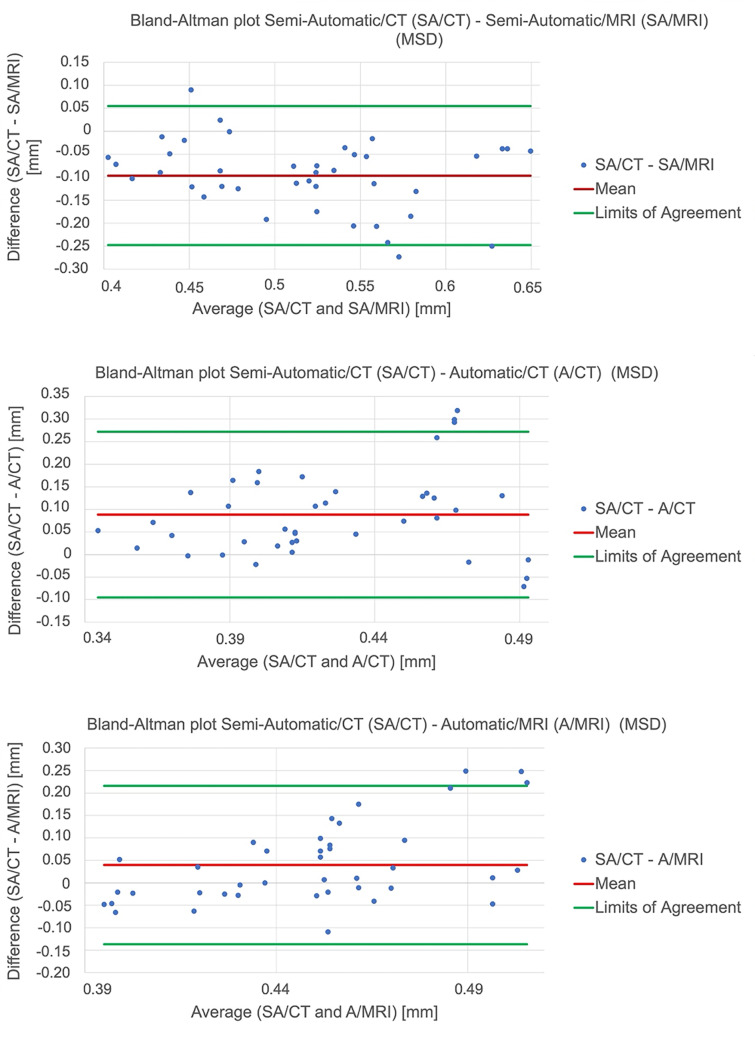
Bland–Altman plots of the differences between the compared combinations of applied segmentation algorithms/modalities and the semi-automatic segmentation of CT data sets with regard to mean surface distances (MSD).

The Bland–Altman analysis indicated high agreement between the compared combinations of the applied segmentation algorithms and modalities with the most common combination, the semi-automatic segmentation of CT data sets, based on the MSD, with 92.5% (37 of 40 differences of the compared measured values of each of the three Bland–Altman plots) within the limits of agreement (Fig 6).

## DISCUSSION

This study highlights two important and interesting aspects. First, T1-weighted MRI-derived data sets enable sufficiently accurate 3D surface models of porcine cadaver models in comparison to a ground truth model. Second, the time-consuming procedure of bone segmentation in MRI-derived images was significantly reduced by applying AI-based segmentation software, while preserving the required overall accuracy. According to the evaluated 3D parameters, this reached the level of known and well-accepted accuracy as it is seen in the commonly applied workflow of CT-derived DICOM data sets and use of licence-based semi-automatic segmentation software, for example, Mimics software. When considering the automatic algorithm, the algorithm examined in this study is not a fully automatic process, as has been tested in other studies.^44^ The applied segmentation algorithm did not need to be trained with preceding data and could therefore be used immediately. Only the already described interactive contributions of the user were needed to let the automatic part run. This had the added advantage that the user himself had some decision-making confidence, but the work could be significantly reduced by ImFusion’s algorithm. Also, although there is little research on the segmentation algorithm of ImFusion or comparable programs based on random walker and watershed segmentation, this was shown in a study by McGrath et al,^22^ but related to other anatomical structures. There, it was described that ImFusion’s algorithm was less time-consuming and physically demanding to use, with the accuracy additionally being improved, compared to manual segmentation. Both advantages could be confirmed in our study with regard to mandibular segmentation.

The focus of MRI imaging was mainly on soft tissue for a long time. In the precise 3D assessment of bony structures (such as the mandibular canal), CT or CBCT still play a primary role. The introduction of new sequences enabled reasonable MRI-based judgement in musculoskeletal imaging including the quantification of cortical and cancellous bone.^7, 21, 35,37^ Consequently, it has become increasingly recognised in the workflow of VSP and in CAD/CAM procedures in recent years as an alternative 3D cross-sectional imaging modality to CT and CBCT, which is not associated with ionising radiation. As previously reported, 3D surface model generation of our T1-weighted MRI sequence revealed comparable accuracy to CT- or CBCT-derived data sets.^24^ In this follow-up study we have included the HD as an additional, critical 3D parameter and compared the segmentation results of two different software solutions. The new insights are of clinical relevance, as the majority of the CAD/CAM workflow for cranio-facial surgery is based on radiation-associated CT- or CBCT-derived data sets. These surgeries also include elective procedures – like orthognathic surgery and dental implant insertion – and procedures in infants – like the treatment of craniosynostosis 25, 40. Patients undergoing these CAD/CAM-assisted procedures are consequently at risk of increased ‘unnecessary’ radiation exposure, although, on the other hand, postoperative results improve by including VSP. Eley et al reported on a novel concept in MRI sequencing to enhance the bone–soft tissue boundary by minimising soft tissue contrast, calling this sequence ‘black bone’.^7–9^ Saarikko et al described their first impressions in preoperative evaluation of patients with craniosynostosis comparing CT with their black bone MRI sequence.^32^ In their study, black bone MRI imaging was equivalent for the evaluation of cranial sutures and intracranial impression, but additionally enabled the assessment of the brain structures in one imaging session. Sychuta et al investigated the accuracy of black bone MRI-derived surgical planning and 3D cutting guide creation for cranial vault remodelling compared with a conventional CT-based workflow.^40^ In this study, comprising ten fresh human cadaver head specimens, they showed that postoperative reconstructions deviated by less than 1.5 mm from the preoperative plan, independently of CT- or MRI-based imaging. They thus demonstrated in a preclinical setting that black bone MRI imaging may be applied for CAD/CAM-assisted calvarial vault surgery with comparable results to the current standard of care.^40^

Virtual surgical planning is also frequently used in mandibular or maxillary reconstruction. Different CAD/CAM solutions have been reported, which are mainly based on radiation-associated imaging modalities.^28^ Sychuta et al also analysed their black bone MRI sequence for mandibular reconstruction with free fibula flap fresh human cadaver specimens. They were able to demonstrate the feasibility of MRI-based VSP and surgical accuracy comparable to CT-based VSP.^41^

Inaccuracies may occur at any step of VSP, but are predominantly attributed to imaging and image processing, including the critical step of segmentation.^18^ Manual segmentation or global thresholding influences the result of segmentation significantly.^46^ Automatic, AI-based solutions may decrease time and inaccuracies, as shown in this study. The AI-based application shown here offered the obvious advantage of saving human resources. On the one hand, this saves time, since the observer only initiated the segmentation and was thus liberated from any tedious edge processing. Consequently, considerably fewer incorrectly marked areas had to be fixed. On the other hand, this circumstance also had an impact on the accuracy of the results, since each additional manual step, which was often necessary with the semi-automatic algorithm, is a potential source of error.

Still limited and heterogenous data are available assessing the trueness, precision and accuracy of MRI-derived 3D bone surface models. We have analysed the fidelity of the 3D surface models in a structured and sound way. ICC coefficients of all 3D parameters (RMSD, MSD, and HD) consistently showed very good intra- and interrater reliability (ICC >0.9). This makes our results relevant and allows further discussion of them. But the comparison of 3D surface models remains difficult, as different parameters and measurements are described in the literature to define ‘accuracy’ in the new digital 3D era. For example, Barr et al were not able to specify a valuable parameter and result for ‘accuracy’ in a recent systematic review and meta-analysis on VSP in mandibular reconstruction.^3^ The reason for this is the heterogeneity of parameters, as the majority of studies compared only 2D linear or angular parameters for fibula segment lengths,^13,16,34,39^ intercondylar distance,^23^ or by comparing interfragmentary gap distances.^38^ In our opinion, this is no longer up to date, which is why our focus in this study has been on pure 3D parameters that encompass the entire porcine cadaver mandibular geometry. This approach seems to be a more contemporary and objective way to compare two similar and relatively complex objects. Within the applied parameters (HD, MSD, and RMSD), HD is the most critical one, as it defines and highlights the worst superimposition of corresponding points of the whole geometry, and RMSD is the most commonly applied in recent literature. In this study, the combination of CT imaging and AI-based segmentation resulted in the best HD (Fig 5a). The RMSD showed for all combinations of imaging modality and segmentation software measurements below 1 mm, which is supposed to be clinically relevant.

Regardless of the fact that MRI protects patients from radiation exposure, it has diagnostic added value compared to CT and CBCT due to its excellent soft tissue depiction, especially in neurocranium imaging or in the staging of soft tissue tumours.^20^ Accordingly, relevant parts of the dental and facial anatomy, such as dental pulp and mucosa or neurovascular structures, skin, and subcutaneous tissues, may be better integrated into the virtual planning process.^1,4,5,17,25,26^ Future studies will need to show how other software solutions, including open-source software, enable feasible and reliable MRI-derived bone models.^48^

### Limitations

First, the study design was based on a purely cadaveric study and may not display and represent results for human mandibles, for example. Nevertheless, the authors believe that this study will add interesting information. Further, comparisons on human mandibles are planned. Second, the used AI-based software is a licence-based solution and may not be available for all potential users.

At the time of the study, only the hand-held scanner with the above-mentioned point accuracy of 50 micrometres was available to the research group. This can already cause a bias in the results, as there are more accurate scanners on the market today. However, this accuracy is sufficient for the segmentation of mandibles, for example, to produce saw templates. Nevertheless, the results presented here reflect an important trend that needs to be followed in the future.

## CONCLUSION

Segmentation of MRI-derived DICOM data sets using a bone-optimised T1-weighted MRI sequence of porcine cadaver mandibles was feasible and generated 3D surface models with comparable accuracy to the corresponding conventional CT-derived DICOM data sets. The use of an AI-based software solution proved to be useful and reliable, and created the desired 3D geometry more quickly while maintaining the necessary quality. CAD/CAM procedures may be planned on the basis of MRI imaging more frequently in the future.

### Acknowledgement

The authors did not receive financial or material support. MRI examinations were covered by in-house funds of the Department of Diagnostic and Interventional Neuroradiology, School of Medicine, Technical University of Munich, Klinikum rechts der Isar, Germany. The authors declare no conflict of interest. The project is not funded.

#### Financial disclosure statement

The authors have nothing to disclose.

#### Data availability statement

The data sets used and/or analysed during the current study are available from the corresponding author on reasonable request.
